# Real time and label free profiling of clinically relevant exosomes

**DOI:** 10.1038/srep30460

**Published:** 2016-07-28

**Authors:** Abu Ali Ibn Sina, Ramanathan Vaidyanathan, Shuvashis Dey, Laura G. Carrascosa, Muhammad J. A. Shiddiky, Matt Trau

**Affiliations:** 1 Centre for Personalized Nanomedicine, Australian Institute for Bioengineering and Nanotechnology (AIBN), Corner College and Cooper Roads (Bldg 75), The University of Queensland, Brisbane QLD 4072, Australia; 2 School of Chemistry and Molecular Biosciences, The University of Queensland, Brisbane, QLD 4072, Australia

## Abstract

Tumor-derived exosomes possess significant clinical relevance due to their unique composition of genetic and protein material that is representative of the parent tumor. Specific isolation as well as identification of proportions of these clinically relevant exosomes (CREs) from biological samples could help to better understand their clinical significance as cancer biomarkers. Herein, we present a simple approach for quantification of the proportion of CREs within the bulk exosome population isolated from patient serum. This proportion of CREs can potentially inform on the disease stage and enable non-invasive monitoring of inter-individual variations in tumor-receptor expression levels. Our approach utilises a Surface Plasmon Resonance (SPR) platform to quantify the proportion of CREs in a two-step strategy that involves (*i*) initial isolation of bulk exosome population using tetraspanin biomarkers (i.e., CD9, CD63), and (*ii*) subsequent detection of CREs within the captured bulk exosomes using tumor-specific markers (e.g., human epidermal growth factor receptor 2 (HER2)). We demonstrate the isolation of bulk exosome population and detection of as low as 10% HER2(+) exosomes from samples containing designated proportions of HER2(+) BT474 and HER2(−) MDA-MB-231 cell derived exosomes. We also demonstrate the successful isolation of exosomes from a small cohort of breast cancer patient samples and identified that approximately 14–35% of their bulk population express HER2.

Exosomes, widely recognised as nano-sized vesicles, represent one of the recently discovered modes of intercellular communication with their ability to transmitting crucial cellular information (e.g., mRNAs, microRNAs and proteins) from parent cell to numerous distant recipient cells^1^,^2^,^3^,^4^. Notably, their unique composition derived from the parent cell and the discovery that cancer cells secrete a larger population of exosomes compared to normal cells has spurred interest in their use as diagnostic markers^5^. This active secretion of exosomes by cancer cells is believed to have numerous functional implications which remain mostly unknown^1^,^6^. Thus, to understand their role in tumor progression, it is critically important to specifically identify the exosomes that cancer cells secrete. Ultimately, analysis of the proportion of clinically relevant exosome subpopulations will significantly improve our ability to diagnose the disease stage as well as devise better therapeutic strategies^6^,^7^,^8^.

The diversity in current conventional bulk isolation methods^9^,^10^ such as ultracentrifugation, filtration, and density gradient separation followed by electron microscopy^11^, ELISA^12^, and western blotting^13^ is a major source of heterogeneity in yield and quality of the isolated exosomes. Importantly, these methods are limited by their ability to accurately isolate exosomes and frequently involve contamination from other vesicles or debris that necessitates subsequent purification steps. Recent progress in microfluidics^14^,^15^,^16^,^17^,^18^ and plasmonic biosensors^19^,^20^,^21^,^22^,^23^,^24^,^25^ have successfully demonstrated the isolation of bulk exosomes as well as exosome subpopulations from biological samples or exosome lysates. Despite their dramatic improvements on isolation, these methods heavily rely on profiling exosomes using either intra-vesicular protein or tumor-specific markers. This strategy however can lead to undesired co-isolation of free proteins or other cellular moieties that also express this targeted marker. In addition, these methods have not yet addressed the quantification of the proportion of this tumor-derived subpopulation with regards to the bulk exosomes population. Identifying proportions of tumor-relevant exosomes could have direct impact with regards to identifying disease state and patient monitoring. For instance, normalising a larger cohort of patients using such an approach can potentially enable simple segregation of patients (*i.e.*, stage of cancer) based on proportions of tumor-specific exosome populations. It is important to note that the variation in the amount of bulk exosomes within a cohort of patients might affect patient stratification based on disease stage^6^. However, analysing proportions of tumor vs healthy exosomes in patients could reduce this variability and potentially improve the diagnosis standards. Further, this can also be extended in monitoring the same patient before and after treatment simply based on the proportions of tumor-relevant exosomes. Thus, there is a need for developing improved isolation strategies to specifically determine and analyse this population of tumor-derived exosomes that can provide insights into their molecular function and role in cancer progression, thereby providing a potential opportunity for non-invasive diagnosis.

Herein, we present a simple and label-free approach for on-chip profiling of CRE among the bulk exosome population isolated from patient serum using a SPR based biosensor. Figure 1A represents the sandwich methodological approach for the detection of CRE. The sandwich strategy combines two subsequent steps: (*i*) capture of bulk population of exosomes present in the sample by targeting a generic exosomal membrane marker, such as CD9 or CD63, which are highly specific to exosomes and also widely expressed in exosomes released by almost all cell types^1^,^26^,^27^, and (*ii*) determine specific populations of CREs among the bulk exosome population using cancer-specific HER2 antibody. We establish proof-of-concept of this approach using breast cancer cell-derived exosomes and subsequently demonstrate the determination of CRE populations from patient serum samples. We use a custom-built SPR platform to capture and detect specific populations of exosomes from these samples. SPR is a powerful analytical approach for immunoaffinity-based assays and analysis of biomolecular interactions with the capability to provide rapid, real-time and label-free read-outs^28^,^29^,^30^,^31^. In SPR, the detection is based on monitoring refractive index changes resulting from the capture of biomolecules onto recognition layer of the metal (typically gold) surface. This capture is associated to a mass increase at the sensing surface over time, which generates a local change in refractive index. Since the refractive index change is directly proportional to the mass change onto the sensing layer, it enables the real-time and label-free readout of the target biomolecules^28^,^29^,^30^,^31^.

## Results

### Exosome Characterization and Analysis

**Figure 1:**
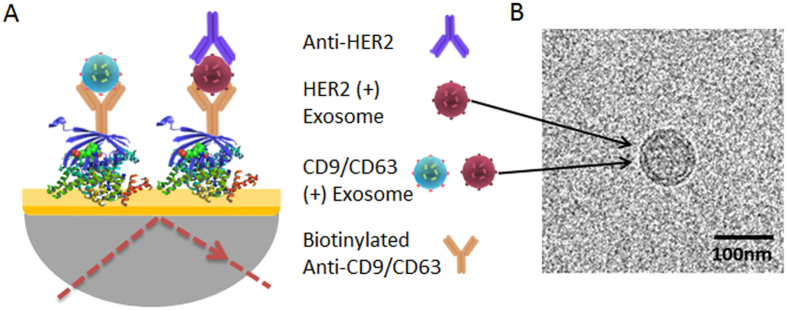
Sandwich approach for the detection of CRE. (**A**) Schematic representation of the sandwich approach for the detection of exosomes in functionalized SPR chip. The chip was first functionalized with biotinylated anti-CD9 or CD63 (Orange) using biotin-avidin chemistry. The exosomes were passed through the SPR machine and captured by the functionalized anti-CD9 and/or anti-CD63 in the SPR chip. The HER2 specific exosomes (Brown) were detected by using anti-HER2 (Violet) (**B**) Cryo-TEM image of the BT474 cell derived exosomes.

To generate breast cancer-specific exosomes, we isolated exosomes from BT474 breast cancer cell-line. This cell line shows overexpression of human epidermal growth factor receptor 2 (HER2), an important breast cancer biomarker and therapeutic target^32^. HER2 expression of the isolated exosomes was verified using western blotting analysis (data not shown). The isolated exosomes were further characterized using cryo-transmission electron microscopy (TEM) (see Fig. 1B and Supporting Information Fig. S1) and Dynamic Light Scattering (DLS) analysis. Cryo-TEM analysis suggested the presence of vesicles with double-wall lipid membrane layers ranging approximately 30–200 nm in diameter. Similarly, DLS analysis suggested that the isolated vesicles were mostly within the size range of 30–300 nm and the average vesicle size was found to be 111 ± 3 nm (Data not shown). These measurements corroborate with previous evidences on exosome characterization^33^,^34^ and suggest the isolated vesicular population to be of exosomal origin. Thus, these isolated exosomes were used for further capture experiments using SPR biosensor.

### Isolation and Detection of Exosomes Using Sandwich Approach

**Figure 2:**
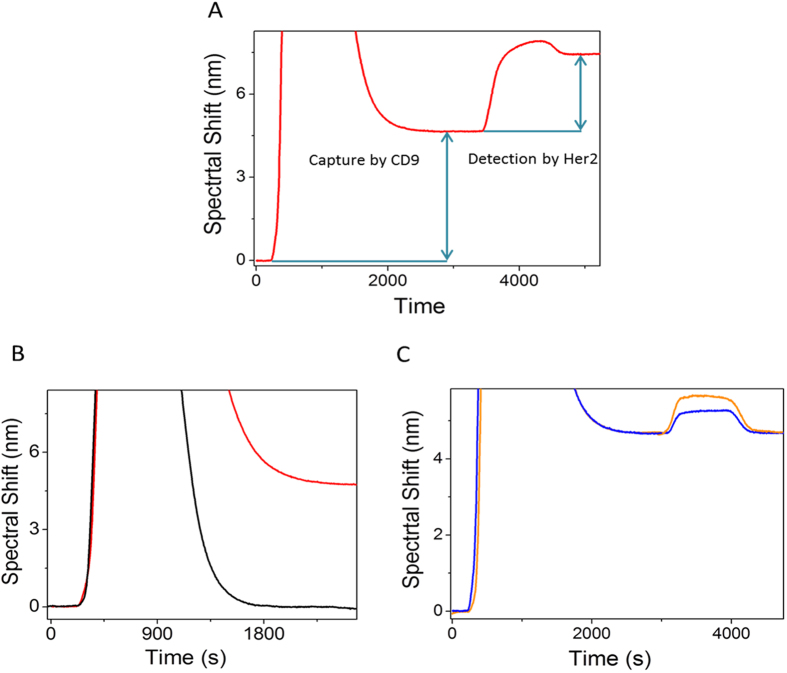
Specificity of the assay. (**A**) SPR signals showing spectral shift generated by the capture of BT474 cell derived bulk exosomes (spiked in 250 μL of PBS) in SPR chip functionalized with anti-CD9 and subsequent detection by anti-HER2 (Red) (**B**) SPR signals showing spectral shift generated by the capture of BT474 cell derived bulk exosomes (spiked in 250 μL of PBS) in SPR chip functionalized with anti-CD9 (Red), without anti-CD9 (Black). (**C**) SPR signals generated by BT474 cell derived exosomes (spiked in 250 μL of PBS) followed by detection with nonspecific anti-PSA (Orange), SPR signal for the capture of MDA-MB-231 cell derives exosomes (spiked in 250 μL of PBS) followed by the detection with anti-HER2 (Blue).

To investigate the utility of our approach in capturing exosome population, BT474 exosomes spiked in PBS (3.3 × 10^4^ exosomes/μL) were driven though the anti-CD9 functionalised SPR sensor. The SPR signal (*i.e.*, SPR spectral shift = 4.75 nm) in Fig. 2A suggests that our method is capable of capturing exosomes from spiked samples. Subsequently, captured exosomes were detected using secondary HER2 antibody driven through the chip. The SPR signal (*i.e.*, SPR spectral shift = 2.75 nm) in Fig. 2A suggests that our approach is capable of detecting HER2 specific exosomes from the isolated bulk exosome population. The lower spectral shift recorded upon detection using HER2 antibody (*i.e.*, SPR spectral shift = 2.75 vs. 4.73 nm) is presumably due to the difference in size of the analytes during capture and detection steps, in this case, exosomes and HER2 antibody. Since SPR signal is associated to the mass increase at the sensing surface, the capture of high molecular weight exosomes in SPR chip gives rise to a higher signal compared to the low molecular weight HER2 antibody attachment during the detection step.

### Specificity of Exosome Capture

To test the specificity of our detection for HER2(+) exosomes, we performed control experiments by (*i*) using SPR chips functionalized with and without anti-CD9 capture antibody, (*ii*) capturing exosomes (3.3 × 10^4^ exosomes/μL) isolated from HER2(−) MDA-MB-231 cell lines and subsequent detection by HER2 detection antibody (*iii*) capturing exosomes (3.3 × 10^4^ exosomes/μL) isolated from HER2(+) BT474 cell lines and subsequent detection using a nonspecific detection antibody (*i.e.*, anti-PSA). In all cases, we observed negligible nonspecific binding of exosomes onto the sensor surface (Fig. 2B,C). These data suggest that our method is specific and selective in capturing and detecting exosomes from spiked samples.

### Analytical Performance of the Sensor

**Figure 3:**
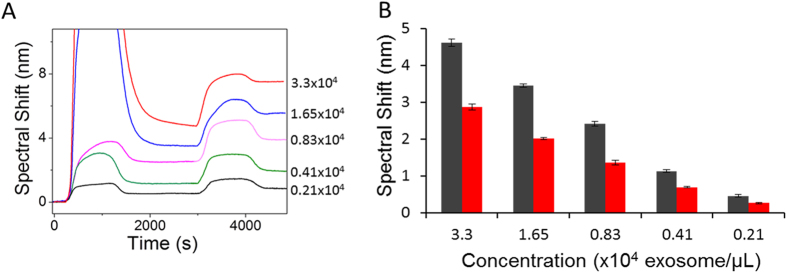
Lower detection limit of the assay. SPR signal showing spectral shift generated by the BT474 cell derived exosomes in different concentration of PBS (3.3 × 10^4^ exosomes/μL (Red), 1.65 × 10^4^ exosomes/μL (Blue), 0.83 × 10^4^ exosomes/μL (Pink), 0.41 × 10^4^ exosomes/μL (Green), 2.07 × 10^3^ (Black) followed by SPR spectral shift generated by HER2 detection antibody. Right panel, corresponding bar chart representing SPR spectral shift generated by the capture of BT474 cell derived exosomes in different concentration of PBS (Red) followed by SPR spectral shift generated by HER2 detection antibody (Black). Each data represents the average of three separate trials (*n* = *3*). Error bars represent the standard deviation of measurements (relative standard deviation (%RSD) was found to be <5% for *n* = *3*).

To investigate the dynamic range and sensitivity of our SPR approach for the capture and detection of exosomes, designated volumes of HER2(+) exosomes were spiked in PBS to obtain desired dilutions (3.3 × 10^4^ exosomes/μL, 1.65 × 10^4^ exosomes/μL, 0.83 × 10^4^ exosomes/μL, 0.41 × 10^4^ exosomes/μL, 2.07 × 10^3^ exosomes/μL) and driven through the anti-CD9 functionalized SPR chip. Dilutions were performed upon obtaining qNano measurements to calculate the approximate concentration of exosomes in each sample. Subsequently, the isolated exosomes were detected using an anti-HER2 antibody. Figure 3 represents the performance of SPR chip for the capture and detection of exosomes from spiked samples with good reproducibility (RSD = <5% for *n* = 3). We found that the average SPR spectral shift ratio (R) between the capture and subsequent detection of exosomes across various tested concentrations is 1.70 (See Supplementary Table S1 for details). This value along with the spectral shift values for bulk exosome population was then used to determine the maximum spectral shift (M_r_) which is the characteristic of the maximum signal that can be obtained for the detection of 100% HER2(+) exosomes. These parameters (R and M_r_) were utilised in our subsequent experiments for the quantification of HER2-specific exosome population from samples containing different proportions of cell derived exosomes and patient samples. Our data also demonstrate that our approach is sensitive enough to detect HER2(+) exosomes from samples containing approximately >2070 exosomes/μL (*i.e.*, 1:4000 in PBS). Further, the linear dynamic range of detection was found to be 2.07 × 10^3^ to 3.3 × 10^4^ exosomes/μL. We observed that at concentrations >3.3 × 10^4^ exosomes/μL, the system was overwhelmed with amount of target exosomes in the sample resulting in signal saturation (see Supplementary Fig. S2). However, this level of detection is comparable with existing immunoaffinity based approaches that rely on microfluidic based isolation and/or plasmonic sensor based read-outs^13^,^14^,^15^,^16^,^17^,^18^,^19^,^20^,^21^,^22^,^23^,^24^,^25^. With the average number of exosomes in biological sample ranging from 1 × 10^5^ to 3 × 10^9^ exosomes/μL^34^,^35^, we believe that our approach (LOD = 2070 exosomes/μL) is potentially suitable for analysing exosomes in clinical applications.

### Isolation of Clinically Relevant Exosomes

**Figure 4:**
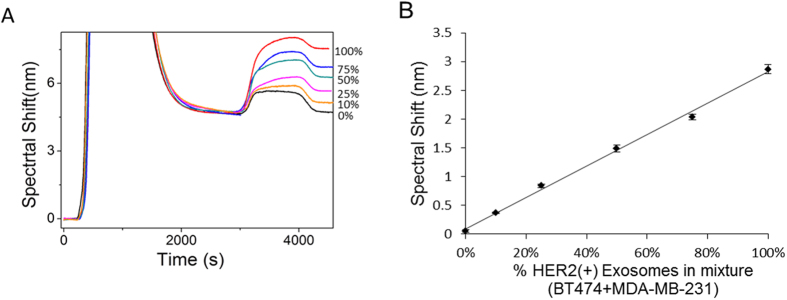
Sensitivity of the assay. SPR signal showing spectral shift generated due to the capture of designated proportions of BT474 (HER2+) and MDA-MB-231 (HER2−) breast cancer cell line (*i.e.*, BT474:MDA-MB-231 at 0:100, 10:90, 25:75, 50:50, 75:25, and 100:0) followed by subsequent quantification of HER2(+) exosomes by HER2 antibody 0% (Black), 10% (Orange), 25% (Pink), 50%(Green), 75% (Blue), 100% (Red). Right panel, corresponding calibration plot. Each data represents the average of three separate trials (*n* = *3*). Error bars represent the standard deviation of measurements (relative standard deviation (%RSD) was found to be <5% for *n* = *3*).

To investigate the ability of our approach in identifying CREs from complex samples, designated proportions of exosomes derived from HER2(+) BT474 (3.3 × 10^4^ exosomes/μL) and HER2(−) MDA-MB-231 (3.3 × 10^4^ exosomes/μL) cell lines (*i.e.*, BT474:MDA-MB-231 = 0:100, 10:90, 25:75, 50:50, 75:25, and 100:0) were spiked in PBS and driven through the anti-CD9 functionalised SPR chip. The SPR spectral shift for the capture of bulk exosomes was found to be almost identical for all the samples. The isolated exosomes were then allowed to interact with the anti-HER2 antibody to identify the proportion of HER2(+) exosomes present in the bulk. As shown in Fig. 4, the SPR spectral shift decreased linearly with decreasing conconcentrations of HER2(+) exosomes in the mixture and the sensor was capable of detecting as low as 10% of spiked HER2(+) exosomes from the isolated bulk exosome population. Further, the percentage of HER2(+) exosomes in these samples were theoretically determined by using equation 1 and 2 (See data analysis in Methods).

### Clinical Sample Analysis

Prior to analysing clinical samples, two control experiments were performed to investigate the specificity of our approach that include: (i) SPR chip functionalized with and without capture CD9 antibody (Supplementary Fig. 3A) and (ii) SPR chips functionalized with CD9 antibody with detection being performed using anti-PSA antibody (Supplementary Fig. 3B). HER2(+) patient sample was driven through the chips and monitored for SPR signals. Notably, in both cases we observed negligible background signal in the capture and detection steps. This indicates that our approach is specific in capturing exosomes of interest from patient samples.

**Figure 5:**
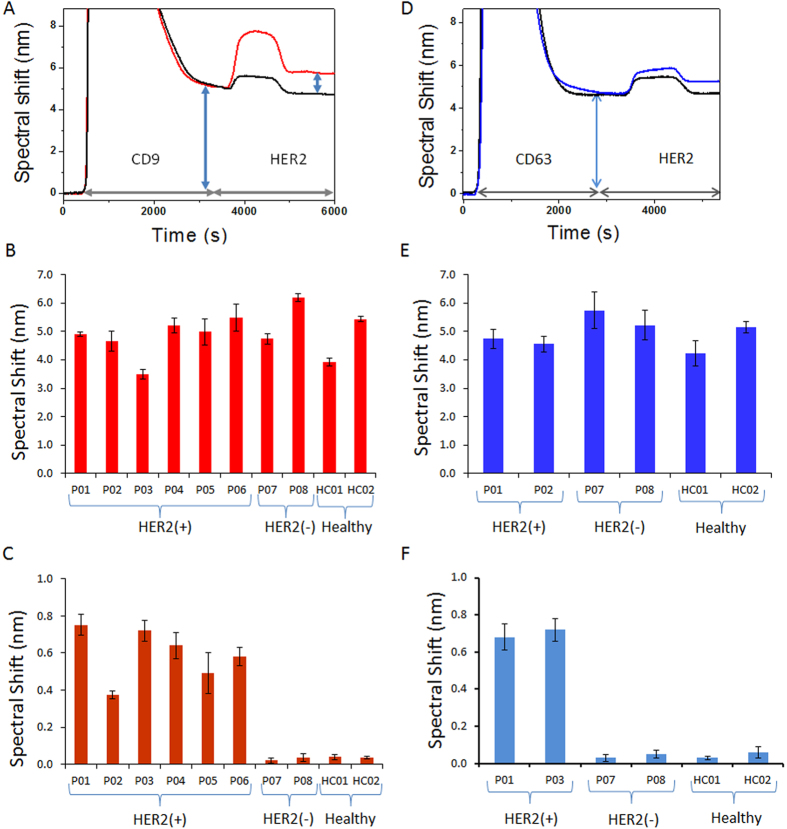
Detection of CRE in patient samples. (**A**) SPR signal showing spectral shift (blue arrows) generated during the capture of exosomes in anti-CD9 functionalized chip followed by detection of HER2(+) exosomes with anti-HER2 in HER2(+) (Red) and HER2(−) patient serum (Black). (B,C) Mean SPR spectral shift for (**B**) capture of bulk exosomes population by anti-CD9 and (**C**) detection of HER2(+) exosomes in six HER2(+) patient, two HER2(−) patient and two healthy control samples. (**D**) SPR signal showing spectral shift (blue arrows) generated during the capture of exosomes in anti-CD63 functionalized chip followed by detection of HER2(+) exosomes with anti-HER2 in HER2(+) (blue) and HER2(−) patient serum (Black). (**E,F**) Mean SPR spectral shift for (**E**) capture of bulk exosomes population by anti-CD63 and (**F**) detection of HER2(+) exosomes in two HER2(+) patient, two HER2(−) patient and two healthy control samples. Each data represents the average of three separate trials (*n* = *3*). Error bars represent the standard deviation of these measurements.

Finally, to demonstrate the diagnostic potential of our approach, we tested the ability of the approach to detect CRE from patient serum samples. CRE populations were identified from six HER2(+) and two HER2(−) breast cancer patient serum samples. Serum samples obtained from two healthy individuals were used as controls. As can be seen in Fig. 5A,B the SPR sensor was effective in isolating bulk exosome population from patient serum samples and healthy controls. This is evident from the similar spectral shifts obtained for all patient and healthy control samples irrespective of their HER2 expression levels. However, the SPR signals obtained for detection (Fig. 5A,C) using anti-HER2 were significantly higher (>10 fold) in case of HER2(+) samples in comparison with HER2(−) and healthy control samples. The SPR spectral data analysis (See data analysis and Supporting Information for details) suggest that the proportion of CRE population present in these HER2(+) serum samples range from approximately 14–35% of the isolated bulk exosome population. This indicates that our approach is effective in identifying and quantifying CREs from patient samples. Further, to confirm the exclusive capture of exosomes and the broad applicability of our method we performed bulk exosome capture experiments using CD63 (Fig. 5D). This is also a vesicular marker widely expressed in exosomes from virtually all tissues and frequently utilised for exosome capture. CRE populations were identified from two HER2(+) and two HER2(−) breast cancer patient serum samples. Serum samples obtained from two healthy individuals were used as controls. As can be seen in Fig. 5E,F a similar trend in capture and detection was observed in comparison to our earlier experiments using CD9 capture. This is evident from the similar spectral shifts obtained for all cancer patient and healthy control samples during capture and detection steps. This indicates that our system performance is consistent across different vesicular markers with no potential bias and can very well be applied for exosome analysis.

## Discussion

A potential challenge in the development of a non-invasive diagnostic assay based on exosome analysis in body fluids is the ability to identify clinically relevant population of exosomes among numerous other exosomes secreted by almost all body cells. Research in the last decade has laid emphasis on developing new methodologies to isolate tumor-specific exosomes from biological samples containing exosomes secreted by almost all cell types. Advances in microfluidics and optical sensing methods have seen new technologies enabling greater robustness and selectivity of exosome targets, with improvements in specificity and limits of detection.

Im *et al*. demonstrated a nano-plasmonic exosome (nPLEX) assay based on transmission surface plasmon resonance through periodic nanohole arrays. Each array functionalized with exosome-specific antibodies enabled profiling of exosome surface proteins and proteins present in exosome lysates^19^. Alternatively, selective targeting of nanoparticles towards cancer cells were utilised as model systems to mimic exosome interaction with cells and these interactions were monitored using molecular dynamic simulations and SPR approach^20^. Similarly, several other immunoaffinity based SPR and microfluidic techniques have been developed for selective screening of tumor derived exosomes^14^,^15^,^16^,^17^,^18^,^19^,^20^,^21^,^22^,^23^,^24^,^25^. Shao *et al*. developed a microfluidic based detection technique utilising a nuclear magnetic resonance system to detect exosomes isolated using magnetic particle tagged antibody^21^. Other microfluidic approaches based on electric field-induced fluid flow^14^, constrained nanochannels^15^,^16^, ciliated micro-pillars^17^ and continuous-flow immunomagnetic bead based separation^18^ have successfully demonstrated the isolation of exosomes using exosomal membrane and/or cancer biomarkers. However, these outlined approaches only considered the isolation of tumor-derived exosomes subpopulations directly from the biological sample (e.g., blood or serum) or from previously purified microvesicles using conventional methods (e.g., ultracentrifugation, exosome isolation kit). In both cases, the presence of other cellular moieties or free proteins expressing the targeted tumor marker might result in a signal bias thereby limiting the identification of CRE subpopulation. This remains a major technological challenge with respect to tumor exosome analysis. Thus, a method to accurately analyse and also identify proportions of these tumor exosomes could have important implications for disease diagnostics and management. For instance, this proportion could potentially inform on the disease stage and stimulate the development of more robust assays by accounting for inter-individual variability on tumor receptor expression levels. In this regard, we developed this sandwich approach to isolate bulk exosome populations using generic exosomal membrane markers and subsequently quantify the proportion of CREs containing tumor-specific information using a tumor-specific detection antibody. In this study we chose HER2 as our tumor-specific biomarker due to its relevance and importance with regards breast cancer diagnosis^36^. For instance, HER2 expression levels have shown noticeable correlation on cancer spread and about 15% to 20% of breast cancer patients demonstrate abnormally high levels of HER2 protein^36^. Thus, with regards to breast cancer diagnosis, it is important to accurately determine HER2 expression level to receive appropriate drug dosage or avoid receiving ineffective drugs if the cancer is deemed to be HER2 negative.

Using this sandwich approach, we developed a model system for the quantification of tumor-derived exosome population among the isolated bulk population. This involved experiments using samples containing designated proportion of exosomes derived from HER2(+) and HER2(−) breast cancer cell lines. Since the total amount of isolated exosomes using anti-CD9 remain the same for each sample, the SPR spectral shift for bulk exosome isolation was found to be similar for all tested samples. However, a linear increase in signal for HER2 detection was observed as a result of increase in the amount of HER2(+) exosomes in the samples. In order to determine the accuracy of detection, the amount of HER2(+) exosomes detected from samples containing designated proportions of HER2(+) and HER2(−) exosomes was then quantified based on theoretical predictions obtained from SPR spectral shifts. The theoretical values for the proportion of target exosomes in each of these samples were obtained using key parameters such as average spectral shift for exosome capture (C_a_) and detection (D_a_), and average spectral shift ratio (R), which is a characteristic of exosome concentration in the sample (see data analysis). It is important to note that the experimental (*i.e.*, spiked proportion) and theoretically determined exosome proportions for each sample were almost identical. This indicates that our approach may potentially be applicable for the quantification of CRE from biological samples.

Finally, we employed this approach for the quantification of CREs from patient serum samples and observed that the SPR sensor was effective (Fig. 5) in identifying the population of CRE’s from the bulk exosome population. The spectral data analysis enabled the quantification of the proportion of exosomes in each of the HER2(+) patient samples and were found to range from 14–35% of the isolated bulk exosome population. The significant difference in signal for HER2(+), HER2(−) and control samples indicate that our approach can clearly distinguish exosomes based on their receptor expression levels. Further, considering the varied expression levels of ubiquitous vesicular markers (*e.g.*, CD9, CD63, CD81 etc.) depending on cell and disease types, the similar levels of CRE populations from patient samples regardless with the use of CD63 or CD9 capture confirms that our approach truly isolates solely exosomes and suggest the broad applicability and reproducibility of our approach. Thus, these data indicate that our approach is suitable for exosome isolation and analysis from patient samples. In addition to this, the ability to clearly distinguish between exosomes based on expression levels holds potential for non-invasive disease diagnosis since current standard diagnosis protocols are based on the identification of receptor expression levels from biopsy samples.

It is important to note that similar SPR signals were observed during CD9 and/or CD63 capture for both cancer and healthy patient samples (Fig. 5B,E). These similar signals observed could be a possible result of (*i*) system saturation due to excessive concentrations of exosomes in the samples (Supplementary Fig. S2), and/or (*ii*) potential balance in number of exosomes in healthy and cancerous conditions due to inhibition of tumor exosome secretion by previously tumor or healthy exosomes in extracellular matrix^6^. This balance might potentially result in patient samples with similar levels of bulk exosomes. Thus, isolation of bulk exosomes alone would not suffice for ideal diagnosis and hence, determining proportions of tumor vs healthy exosomes from patient samples could have much more significance compared to bulk analysis approaches. Further, with the average number of exosomes in biological sample ranging from 1 × 10^5^ to 3 × 10^9^ exosomes/μL^33^,^34^, our system performance is very much sensitive to detect majority of exosomes from patient samples. Thus, we believe this proof-of-concept approach for the detection performance of HER2-specific exosomes in clinical samples is specific and offers potential for clinical diagnosis. We believe further optimization to the protocol and device geometry (*e.g.*, length, width, and height of sensing surface) can substantially improve the system performance to achieve a broader dynamic range. Although the presented approach specifically focused on breast cancer, screening of exosomes based on a larger panel of tumor markers using this approach could establish a more comprehensive framework for broad analysis of exosomes from different cancer types.

In conclusion, we have developed a simple method for the quantification of the proportion of CRE’s from complex biological fluids. With several immune-affinity based approaches developed over the years, we believe our approach provides two critical improvements to existing approaches for exosome isolation: (*i*) quantification of the proportion of tumor-specific exosome subpopulations with respect to bulk exosome population, (ii) ability to identify tumor-specific exosomes on-chip avoids the need for downstream analysis, leading to an easier and rapid approach. We have demonstrated that our approach is sensitive to detect approximately 2070 exosomes/μL from breast cancer cell-derived exosome samples and identified the presence of approximately 14–35% of tumor-specific exosomes from breast cancer patient serums. We believe that the approach is not just limited to HER2 specific exosome detection and can be extended towards identification of multiple tumor-specific exosome populations from a diverse range of tumor types. In addition to this, we predict that our approach could have broad applications in the field of cancer research and with further improvements, can potentially find its relevance as a simple diagnostic tool during cancer diagnosis and treatment.

## Methods

### Cell culture and isolation of exosomes

Breast cancer (HER2(+): BT-474; HER2(−): MDA-MB-231) and prostate cancer (PSA(+): PC3) cell lines were maintained in microvesicles depleted serum free Media 171 (Gibco, UK) supplemented with Mammary Epithelial supplement (Gibco, UK), 1% Penicillin/streptomycin and grown in 5% CO_2_ at 37 °C. The conditioned medium from 10^6^ cells was collected after 60 h and centrifuged at 2000 × g for 30 min to eliminate cell contamination (*e.g.*, cells and debris). Exosomes were isolated using Total Exosome isolation reagent (Life Technologies) as per manufacturer’s instructions. Briefly, the supernatant was transferred to a new tube and the isolation reagent was added to the tube in the ratio 2:1. The samples were incubated overnight at 4 °C followed by filtration using 0.22 μm filter and centrifugation at 10000 × g for 1 h to obtain exosome pellets. Exosome pellets were then resuspended in 100 μL PBS (10 mM, pH 7.0) and stored at −20 °C for further use.

### Cryo-transmission electron microscopy (cryo-TEM) and dynamic light scattering analysis

For cryo-TEM, 4 μL of exosome preparations were directly adsorbed onto lacey carbon grids (Quantifoil, Germany) and plunged into liquid ethane, using an FEI Vitrobot Mark 3 (FEI Company, The Netherlands). Grids were blotted at 100% humidity at 4 °C for about 3–4 s. Frozen/vitrified samples were imaged using Tecnai T12 Transmission Electron Microscope (FEI Company) operating at an acceleration voltage of 120 kV. Images were taken at 30,000x magnification, (approximate dose of 13.6 electrons/Å2), using an FEI Eagle 4k CCD (FEI Company), and Serial EM image acquisition software. For dynamic light scattering method, please see Supporting Information of ref. 14.

### Device functionalization

All experiments were performed in a custom-made SPR platform^30^. Initially, the SPR sensor chips (5 nm Ti and 50 nm Au) were cleaned by rinsing with hot acetone, ethanol and deionised water and dried under flow of nitrogen gas. Subsequently, the chips were dipped in piranha solution (70% H_2_SO_4_–30% H_2_O_2_) for few seconds, rinsed with water and dried under flow of nitrogen gas. Following this step, the chip was placed in the SPR platform and PBS buffer was flowed in continuous at a flow rate of 0.6 mL/h. Then, 250 μL samples of biotinylated BSA (100 μg/mL in PBS, Invitrogen) were directly injected in the SPR flow system at the same flow rate. To remove nonspecific adsorption on the gold surface, 3% BSA was used to cover the remaining empty space of the SPR gold chip. After that, streptavidin (100 μg/mL in PBS, Invitrogen) was run through the channel to couple with the functionalised biotinylated BSA. The biotinylated anti-CD9 and/or CD63 were then conjugated with the streptavidin to capture exosomes. The HER2 antibody is then driven through the SPR chips to detect tumor specific exosomes. The SPR signals were monitored using custom-made Labview software.

### Exosome capture and detection

The concentration of exosomes in the isolated pellets was obtained using qNano measurements performed as described previously^37^. Concentration measurements were obtained by calibrating particle count rate recordings against a reference particle suspension (polystyrene beads, *d* = 115 nm). Samples were prepared by spiking designated volumes of isolated exosomes in PBS (1 mM, pH 7.0) to obtain the desired sample dilutions (1:250 to 1:4000) (Table 1). Serum samples (1 mL) of breast cancer patients and healthy individuals were obtained from Ventyx Wesley Research Institute Tissue Bank, Brisbane, Australia under the UQ HREC ethical approval number 2011001315 and Bellberry application number 2015-12-817. All the serum samples were stored in −80°C until further use. Immunohistochemical expression analysis suggested overexpression (3+: P01–P06) and very low expression (1+; P07, P08) of HER2 in these patient serums. All the exosome containing target samples were passed through the SPR system to capture the exosomes in previously functionalised SPR chip. Finally, the HER2 (+) exosomes were then detected by running anti-HER2 through the SPR system.

### Data analysis

The average spectral shift ratio (R) between capture and detection of exosomes was obtained from the ratios of the SPR spectral shifts obtained during capture step using anti-CD9 (C_a_) and detection step using anti-HER2 (D_a_) across various tested concentrations (See Table S1 in Supporting Information). The theoretical percentage of HER2(+) exosomes present in a given sample (e.g., designated mixture of BT474 and MDA-MB-231cell-derived exosomes or patient samples) was then calculated by using equation 1 and 2 (see also Tables S2 and S3 in Supporting Information).


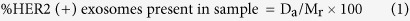


D_a_ = SPR Spectral Shift for the detection of exosomes using anti-HER2


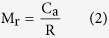


where, M_r_ = Maximum SPR Spectral Shift could possibly be achieved during the read-out step using anti-HER2 (*i.e.*, for a sample containing 100% of HER2+ exosomes)

C_a_ = Average SPR Spectral Shift for the capture of exosomes

R = 1.70 (Average Ratio between capture and detection signals obtained for HER2+ cell derived exosomes at various tested concentrations).

## Additional Information

**How to cite this article**: Sina, A. A. I. *et al*. Real time and label free profiling of clinically relevant exosomes. *Sci. Rep.* **6**, 30460; doi: 10.1038/srep30460 (2016).

## Supplementary Material

Supplementary Information

## Figures and Tables

**Table 1:** Concentration of exosomes in the isolated pellet measured by qNano.

Sample Dilution in PBS	Concentration of the sample (no. of exosomes/μL)
1:250	3.3 × 10^4^
1:500	1.65 × 10^4^
1:1000	0.83 × 10^4^
1:2000	0.41 × 10^4^
1:4000	2.07 × 10^3^
