# Supplementary material for: Real time and label free profiling of clinically relevant exosomes
Source: Sci Rep. 2016 Jul 28;6:30460. doi: 10.1038/srep30460 (PMC4964344; doi:10.1038/srep30460)
Supplement: Supplementary Information [file srep30460-s1.docx]

**Supplementary Information**

**for**

Real time and label free profiling of clinically relevant exosomes

*Abu Ali Ibn Sina,^a✝^ Ramanathan Vaidyanathan,^a✝^ Shuvashis Dey,^a^ Laura G. Carrascosa,^a^* Muhammad J. A. Shiddiky^a^** ^ξ^ *and Matt Trau^a,b^**

*^a^Centre for Personalized Nanomedicine, Australian Institute for Bioengineering and Nanotechnology (AIBN), Corner College and Cooper Roads (Bldg 75), The University of Queensland, Brisbane QLD 4072, Australia*

*^b^School of Chemistry and Molecular Biosciences, The University of Queensland, Brisbane, QLD 4072, Australia*

**Email: lgcarrascosa@uq.edu.au (LGC); m.shiddiky@griffith.edu.au (MJAS);* [*m.trau@uq.edu.au*](mailto:m.trau@uq.edu.au) *(MT)*

Tel: +61-7-33464178; Fax: +61-7-33463973

*^✝^Authors contributed equally*

***^ξ^*** *Present Address: School of Natural Sciences, Griffith University (Nathan Campus), Nathan, QLD 4111, Australia*

**Data Analysis**

**Table S1| Average ratio of the SPR spectral shift for the capture and detection of exosomes**

| Concentration of the sample  (exosomes/µL) | Average SPR spectral shift for the capture of exosomes | Average SPR spectral shift for the detection of HER2(+) exosomes | Ratio between capture and detection signal | Average ratio (R) |
| --- | --- | --- | --- | --- |
| 3.3🞨10^4^ | 4.62 | 2.75 | 1.73 | 1.70 |
| 1.65🞨10^4^ | 3.45 | 2.06 | 1.71 |  |
| 0.83🞨10^4^ | 2.41 | 1.45 | 1.72 |  |
| 0.41🞨10^4^ | 1.14 | 0.72 | 1.65 |  |
| 2.07🞨10^3^ | 0.47 | 0.3 | 1.70 |  |

**Table S2| Analysis of cell derived samples**

| HER2(+) exosomes in mixture (Experimental) | Average SPR Spectral Shift for the Capture of Bulk Exosomes (C_a_) | Average SPR spectral shift for HER2(+) Exosome detection (D_a_) | %HER2(+)  exosomes in mixture (Theoretical) |
| --- | --- | --- | --- |
| 0% | 4.73 | 0.05 | 1.80% |
| 10% | 4.77 | 0.37 | 13.02% |
| 25% | 4.75 | 0.84 | 29.92% |
| 50% | 4.65 | 1.49 | 54.35% |
| 75% | 4.66 | 2.04 | 74.13% |
| 100% | 4.75 | 2.87 | 102.35% |

**Table S3| Patient Sample analysis**

| Patient Sample | Average SPR Spectral Shift for the Capture of Bulk Exosomes (C_a_) | Average SPR Spectral Shift for the Detection of HER2 (+) Exosomes by anti-HER2 (D_a_) | % HER2 (+) exosomes present in Sample |
| --- | --- | --- | --- |
| P1 | 4.91 | 0.75 | 25.95 |
| P2 | 4.65 | 0.38 | 13.92 |
| P3 | 3.48 | 0.72 | 35.12 |
| P4 | 5.20 | 0.64 | 20.98 |
| P5 | 4.98 | 0.49 | 16.72 |
| P6 | 5.48 | 0.58 | 18.01 |





**Figure S1|** **Exosome Characterization**. TEM image of exosome population derived from BT474 breast cancer cell line.


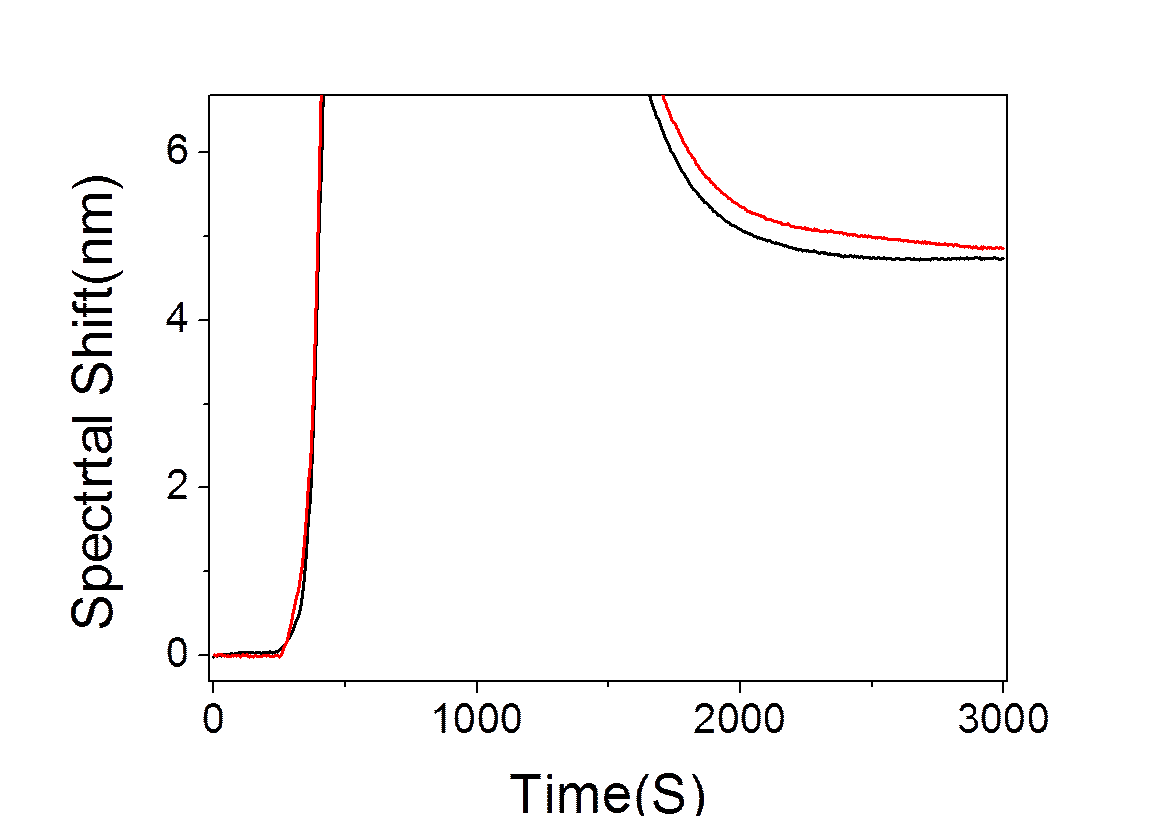


**Figure S2|** **Effect of concentration and SPR signal saturation**. SPR signal showing spectral shift generated by the BT474 cell derived exosomes at concentrations of 3.3🞨10^4^ exosomes/ µL (Black) and 8.25🞨10^4^ exosomes/ µL (Red), respectively.


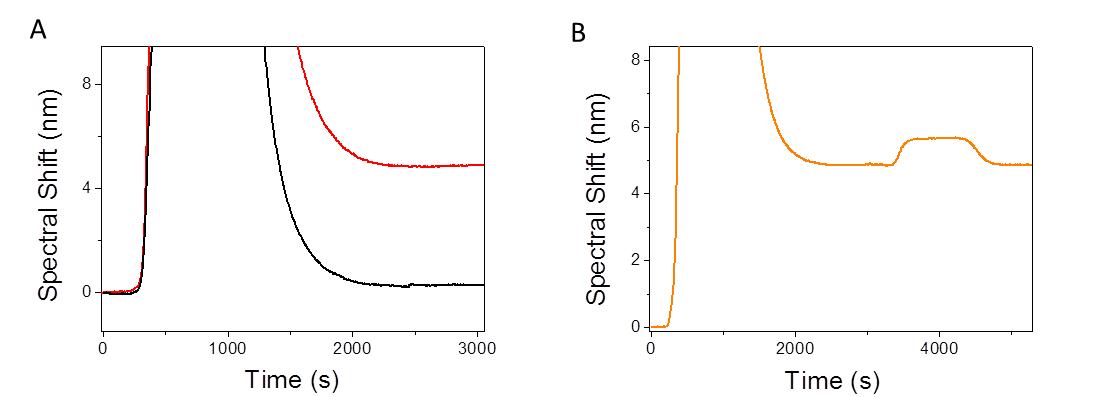


**Figure S3|** Control study for patient samples. (A) SPR signal showing spectral shift generated by the HER2(+) serum sample over the SPR chip functionalised with CD9(Red) and without CD9 (Red).(B) SPR signal showing spectral shift generated during the capture of exosomes in anti-CD9 functionalized chip followed by detection with Anti-PSA.
